# HoloFoodR: a statistical programming framework for holo-omics data integration workflows

**DOI:** 10.1093/bioinformatics/btaf605

**Published:** 2025-11-09

**Authors:** Tuomas Borman, Artur Sannikov, Robert D Finn, Morten Tønsberg Limborg, Alexander B Rogers, Varsha Kale, Kati Hanhineva, Leo Lahti

**Affiliations:** Department of Computing, University of Turku, Turku 20014, Finland; Department of Life Technologies, University of Turku, Turku 20014, Finland; Wellcome Genome Campus, EMBL-EBI, Hinxton, Cambridgeshire CB10 1SA, United Kingdom; Center for Evolutionary Hologenomics, GLOBE Institute, Faculty of Health and Medical Sciences, University of Copenhagen, Copenhagen 1353, Denmark; Wellcome Genome Campus, EMBL-EBI, Hinxton, Cambridgeshire CB10 1SA, United Kingdom; Wellcome Genome Campus, EMBL-EBI, Hinxton, Cambridgeshire CB10 1SA, United Kingdom; Department of Life Technologies, University of Turku, Turku 20014, Finland; Department of Computing, University of Turku, Turku 20014, Finland

## Abstract

**Summary:**

Holo-omics is an emerging research area that integrates multi-omic datasets from the host organism and its microbiome to study their interactions. Recently, curated and openly accessible holo-omic databases have been developed. The HoloFood database, for instance, provides nearly 10 000 holo-omic profiles for salmon and chicken under controlled treatments. However, bridging the gap between holo-omic data resources and algorithmic frameworks remains a challenge. Combining the latest advances in statistical programming with curated holo-omic data sets can facilitate the design of open and reproducible research workflows in the emerging field of holo-omics.

**Availability and implementation:**

HoloFoodR R/Bioconductor package and the source code are available under the open-source Artistic License 2.0 at the package homepage https://doi.org/10.18129/B9.bioc.HoloFoodR.

## Introduction

The rapid advancement of omics technologies, including (meta)genomics and metabolomics, has been driven by breakthroughs in computational methods (Moreno-Indias *et al.* 2021, Marcos-Zambrano *et al.* 2023, Santamaria and Pinto 2024). These developments have enabled holo-omics, a rapidly emerging field that uses an integrative approach to comprehensively collect and analyse omic data from both a host organism and its associated microbiome, collectively referred to as the *holobiont* (Nyholm *et al.* 2020, Limborg *et al.* 2018, Odriozola *et al.* 2024). The holo-omic approach has improved our understanding of complex biological systems, for example, in aquaculture (Limborg *et al.* 2018, Brealey *et al.* 2024).

Holo-omic research and the collaborative development of computational methods could benefit from curated, open-access data resources. The limited availability of comprehensive multi-omic data resources can impede research progress, constraining computational method development. Open data portals with an accessible Application Programming Interface (API) are crucial in overcoming these challenges. They facilitate research by providing access to data resources and supporting the collaborative development and benchmarking of new data science methods to extract insights from large, curated datasets (Pasolli *et al.* 2017).

Data can be limited in value unless it adheres to the FAIR principles (Findability, Accessibility, Interoperability, Reuse) (Wilkinson *et al.* 2016). API interfacing and data wrangling require specialized skills, leading to non-transferable, error-prone workflows not easily reusable by the wider scientific community. Therefore, standardised open-source workflows are needed to search, retrieve, and convert data into a suitable format for downstream analyses. To narrow the gap between upstream data retrieval and downstream analysis, we developed the HoloFoodR package, which facilitates seamless programmatic linking of the holo-omic data via the HoloFood data portal API and analysis methods from the Bioconductor (Gentleman *et al.* 2004, Callahan et al. 2016).

## Materials and methods

The HoloFood data portal is an open-access portal of curated holo-omic data and analyses, developed by the international HoloFood consortium (Rogers et al. 2025) and hosted by the European Bioinformatics Institute (EMBL-EBI). It centralizes access to heterogeneous data resources, including the European Nucleotide Archive for sequence data, MGnify for metagenomic data, and MetaboLights for metabolomic data, and tracks their interrelations via a web portal and free API, covering nearly 10 000 samples from over 2000 individual chickens and salmon. Biomolecular and physiological measurements were collected at the level of each individual specimen in the project to explore the effects of novel sustainable feeds on physiological processes in farmed animals. In addition, the metadata of each sample is stored in the EMBL-EBI BioSamples service (Courtot et al. 2019).

We developed a conceptual framework to support best practices of statistical programming in holo-omic research and implemented the work as the HoloFoodR R software library. The package relies on specialized data containers that have been designed to organize the multi-omic data in a structured format. Once the data have been imported into these formats, users can leverage state-of-the-art statistical programming methods for data processing and analysis.

### Integrative data containers

Data containers provide structured, standardized storage for data, proving useful in life science informatics where complex, hierarchical, and multi-source data collections are common to describe the studied phenomenon (Drnevich et al. 2025). Custom data containers simplify handling diverse omics data, supporting the design of modular and reproducible workflows.

Two data structures are central to our approach, encompassing the need to organize data for a single omics type, and subsequently link these across multiple omics. First, the TreeSummarizedExperiment (TreeSE) data container (Huang et al. 2020) standardizes single-omic data, such as metagenome or metabolome-derived data sets. It stores experimental data, along with feature and sample metadata, in a structured format. Additionally, it supports the integration of hierarchical information on the features and samples, such as phylogenetic trees. The second container, MultiAssayExperiment (MAE) (Ramos et al. 2017), acts as a logical complement by integrating multiple, heterogeneous omics datasets, seamlessly linking samples across single-omic datasets. Together, these data containers form the basis for an ecosystem of interoperable methods developed by the Bioconductor community.

### HoloFoodR workflow

**Figure 1.**
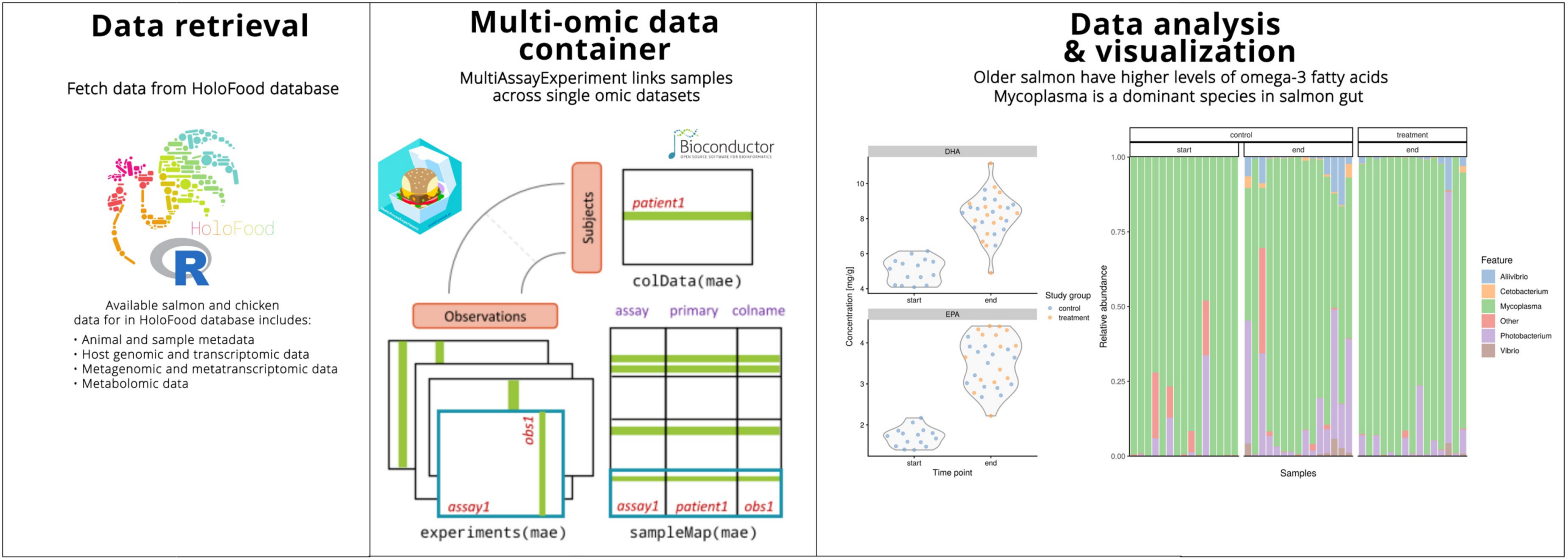
Data science workflow for holo-omic analysis. Programmatic access to open omic databases is facilitated by the dedicated software that feeds the data to R computational environment. The Bioconductor packages are utilised to visualise microbial genera abundances and to analyse time effect on omega-3 fatty acid concentrations in salmon muscles. The illustration and logo of the multi-assay data container have been adopted from the MultiAssayExperiment vignette (Artistic License 2.0).

The HoloFoodR package structures data in these single and multi-omic formats, enabling efficient data importing from holo-omic databases for streamlined analysis and integration. The HoloFoodR workflow is summarized in Figure 1. The package uses six functions to query and retrieve data from the HoloFood and MetaboLights databases (Table 1). A key feature is its ability to organize versatile data combinations into the MAE data container. Thus, it transforms complex data from the HoloFood API into a standardized structure for downstream statistical analyses in the Bioconductor data science environment (Ramos et al. 2017).

**Table 1.** HoloFoodR functions that facilitate data search and retrieval.

Function	Description
addMGnify()	Integrate the results retrieved with getResult() with the metagenomic datasets fetched using the MGnifyR package. The result is in MultiAssayExperiment format.
doQuery()	Search the HoloFood database for animals, genome catalogues, samples, or viral catalogues.
getData()	Retrieve diverse data types from HoloFood database. Returns a list or data.frame, depending on the query. Offers more flexibility than getResult(), but returns unstandardised data structures.
getMetaboLights()	Retrieve processed metabolomic data from MetaboLights as list or TreeSummarizedExperiment format.
getMetaboLightsFile()	Downloads raw metabolomic data files from MetaboLights.
getResult()	Retrieve sample-level data (e.g. metadata and measurements) from the HoloFood database in MultiAssayExperiment format.

Furthermore, HoloFoodR leverages the MetaboLights database to retrieve metabolomic datasets indicated by project identifiers in the HoloFood data. The data can then be integrated with complementary metagenomic data resources that are available via the MGnify database using the MGnifyR package (Borman et al. 2024).

### Bioconductor methods ecosystem

When data from the HoloFood database is retrieved via the HoloFoodR package, users gain direct access to single- and multi-omic downstream analysis methods available in Bioconductor. It currently offers 2300 R packages, developed by a diverse community of individual contributors for a wide range of fields (see, e.g. Gentleman et al. 2004, Callahan et al. 2016, Amezquita et al. 2020, Drnevich et al. 2025). Bioconductor’s mia framework centres around these containers and supports a range of analysis and visualisation methods for microbiome research (Borman et al. 2025). Similarly, the metabolomic data can be pre-processed, analyzed and visualized using the notame package (Klåvus et al. 2020).

## Results

This section presents a practical use case of applying HoloFoodR in a holo-omics workflow. The workflow includes the following steps:

1. Fetch and integrate data from the HoloFood and MGnify databases.
2. Filter, clean, and transform data for analysis.
3. Explore and summarize the data.
4. Test associations between fatty acids, time, and treatment.
5. Test associations between microbiome composition, time, and treatment.
6. Characterize the joint variation between the parallel omics measurements.

The full workflow, including figures and data refinement, is available in the package vignette at https://ebi-metagenomics.github.io/HoloFoodR/articles/case_study.html.

### Data import and wrangling

The workflow begins by querying the HoloFood database for available animals. We gathered all salmon entries and their metadata to explore data types and samples. These samples were compiled into an MAE data container. For metagenomic data, we leveraged the capabilities of the MGnifyR package to identify the sample IDs from the MGnify database and fetch the metagenomic data as an MAE object. The consistent data format supports seamless merging of HoloFood and MGnify datasets. The resulting container holds various types of omics with associated metadata for downstream analyses.

Using the MAE object for data wrangling, we focused on salmon from Trial A in the HoloFood database to study the effects of fermented seaweed as a feed additive. Samples were obtained from animals euthanized at either the start or end of the trial. Fatty acid concentrations were measured in muscle tissue, while metagenomic samples were taken from the intestine.

The mia framework (Borman et al. 2025) provides an intuitive R interface designed for the downstream analysis, especially for microbiome data. We filtered and agglomerated the data to focus on specific microbes and fatty acids, applying centred log-ratio transformation to metagenomic data and logarithmic transformation to fatty acid data. The full details are provided in the HoloFoodR package vignette.

### Downstream analysis

After data retrieval and basic exploration, we examined the effects of seaweed treatment and ageing. No treatment impact on fatty acid composition was observed, consistent with the recent HoloFood study (Rasmussen et al. 2025). However, we found temporal effects: as the salmon grew, concentrations of several fatty acids (docosahexaenoic, eicosapentaenoic, linoleic, oleic, palmitic, and stearic) increased (see Figure 1, data analysis panel, which highlights the first two fatty acids).

Microbial community analysis indicated a dominance of *Mycoplasma*, consistent with previous studies (Zarkasi et al. 2014, Bozzi et al. 2021, see Fig. 1, data analysis panel). We also observed an increase in microbial diversity (Shannon index) with time, confirmed by Principal Coordinate Analysis (Bray-Curtis dissimilarity).

To explore the co-abundance of fatty acids and microbial genera, we performed multi-omic factor analysis (Argelaguet et al. 2020), revealing that *Cetobacterium*, *Vibrio*, *Aliivibrio*, and *Photobacterium* covaried with overall fatty acid levels, while *Mycoplasma* showed no such correlation.

## Discussion and conclusions

Integrating multiple omics layers provides holistic insights into complex systems. Holo-omics is a new field that emphasises the interactions between the hosts and their microbiomes. However, such analyses rely on curated data, computational environments, and algorithmic tools. Collaborative development and sharing in holo-omics can advance the field and prevent redundant efforts (Shetty and Lahti 2019; Lahti 2018).

To fill the gap between holo-omic data and downstream analysis methods, we developed the HoloFoodR package. Our work offers programmatic access to the HoloFood data portal and integrates with complementary data sources and Bioconductor analysis tools, supporting open data science in holo-omics.

HoloFoodR simplifies data retrieval by minimizing external data wrangling and adhering to standardized data structures that support efficient use of the multi-omic analyses methods. This standardization replaces lengthy *ad-hoc* code with concise and tested open-source solutions. We demonstrate this approach with an end-to-end workflow, highlighting the use of multi-assay data containers to facilitate access to Bioconductor methods.

Beyond method development and analysis, HoloFood data can aid in teaching multi-omics techniques. HoloFoodR offers simplified access to real-world omic data, enabling more proficient users to “learn by doing.” The complexity of this data makes it superior to “toy” datasets for teaching advanced data cleaning and analysis skills (Drnevich et al. 2025).

The HoloFood data portal currently provides the interconnectivity between the samples and omics datasets, based on BioSamples identifiers. This linkage provides a template for future multi-omics datasets, and as the number of datasets increases, the HoloFoodR package could be generalised so that the concepts can be readily applied to other datasets.

Despite the benefits, there are also limitations. First, raw spectral metabolite data from the MetaboLights database requires extensive preprocessing, often with external tools (Klåvus et al. 2020). Whereas the proposed custom data structures can readily support downstream analyses, their construction and use require sufficient R programming skills.

Thus, the methods and open data science strategy that we have proposed can serve as a template for conducting multi-omic analyses. The HoloFoodR package can be adapted for other data resources relevant to holo-omic research, where the growing Bioconductor ecosystem offers an expanding set of data analysis tools.

## Data Availability

Available in the package vignette https://ebi-metagenomics.github.io/HoloFoodR/articles/case_study.html.
